# Sensing in the dark: Constructive evolution of the lateral line system in blind populations of *Astyanax mexicanus*

**DOI:** 10.1002/ece3.11286

**Published:** 2024-04-23

**Authors:** Roberto Rodríguez‐Morales

**Affiliations:** ^1^ Department of Anatomy & Neurobiology, School of Medicine University of Puerto Rico San Juan Puerto Rico

**Keywords:** adaptation, cavefish, evolution, hair cells, lateral line, sensory systems

## Abstract

Cave‐adapted animals evolve a suite of regressive and constructive traits that allow survival in the dark. Most studies aiming at understanding cave animal evolution have focused on the genetics and environmental underpinnings of regressive traits, with special emphasis on vision loss. Possibly as a result of vision loss, other non‐visual sensory systems have expanded and compensated in cave species. For instance, in many cave‐dwelling fish species, including the blind cavefish of the Mexican tetra, *Astyanax mexicanus*, a major non‐visual mechanosensory system called the lateral line, compensated for vision loss through morphological expansions. While substantial work has shed light on constructive adaptation of this system, there are still many open questions regarding its developmental origin, synaptic plasticity, and overall adaptive value. This review provides a snapshot of the current state of knowledge of lateral line adaption in *A. mexicanus*, with an emphasis on anatomy, synaptic plasticity, and behavior. Multiple open avenues for future research in this system, and how these can be leveraged as tools for both evolutionary biology and evolutionary medicine, are discussed.

## Graphical abstract

Previous studies predominantly investigated eye regression in cavefish evolution, but less is known about constructive evolution of non‐visual sensory systems in subterranean species. This review highlights our state of knowledge on mechanosensory lateral line expansions in *Astyanax mexicanus*, emphasizing anatomical features, synaptic plasticity, and behavior, while proposing future research directions in both evolutionary biology and evolutionary medicine.

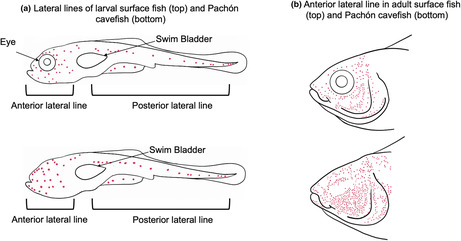

## 1 INTRODUCTION

Animals have colonized diverse habitats all over the world, evolving a stunning display of adaptations to survive under extreme environments. For example, Alaskan wood frogs freeze their bodies to survive low temperatures during the winter (Larson et al., 2014), kangaroo rats can survive in the desert with almost no water (Tappe, 1941), and giant tubeworms live on the floor of the Pacific Ocean under freezing temperatures, while tolerating extremely high hydrogen sulfide concentrations (Bright & Lallier, 2010). Similar to hostile conditions in the darkness of the deep sea, but closer to human interaction, are troglobite species that live under the perpetual darkness of cave habitats (Culver, 1982). A surprising diversity of invertebrates can be found within caves, including snails, worms, spiders, beetles, and shrimps, and most of these evolved a similar constellation of traits that allowed for successful cave colonization (Culver & Pipan, 2019).

**FIGURE 1:**
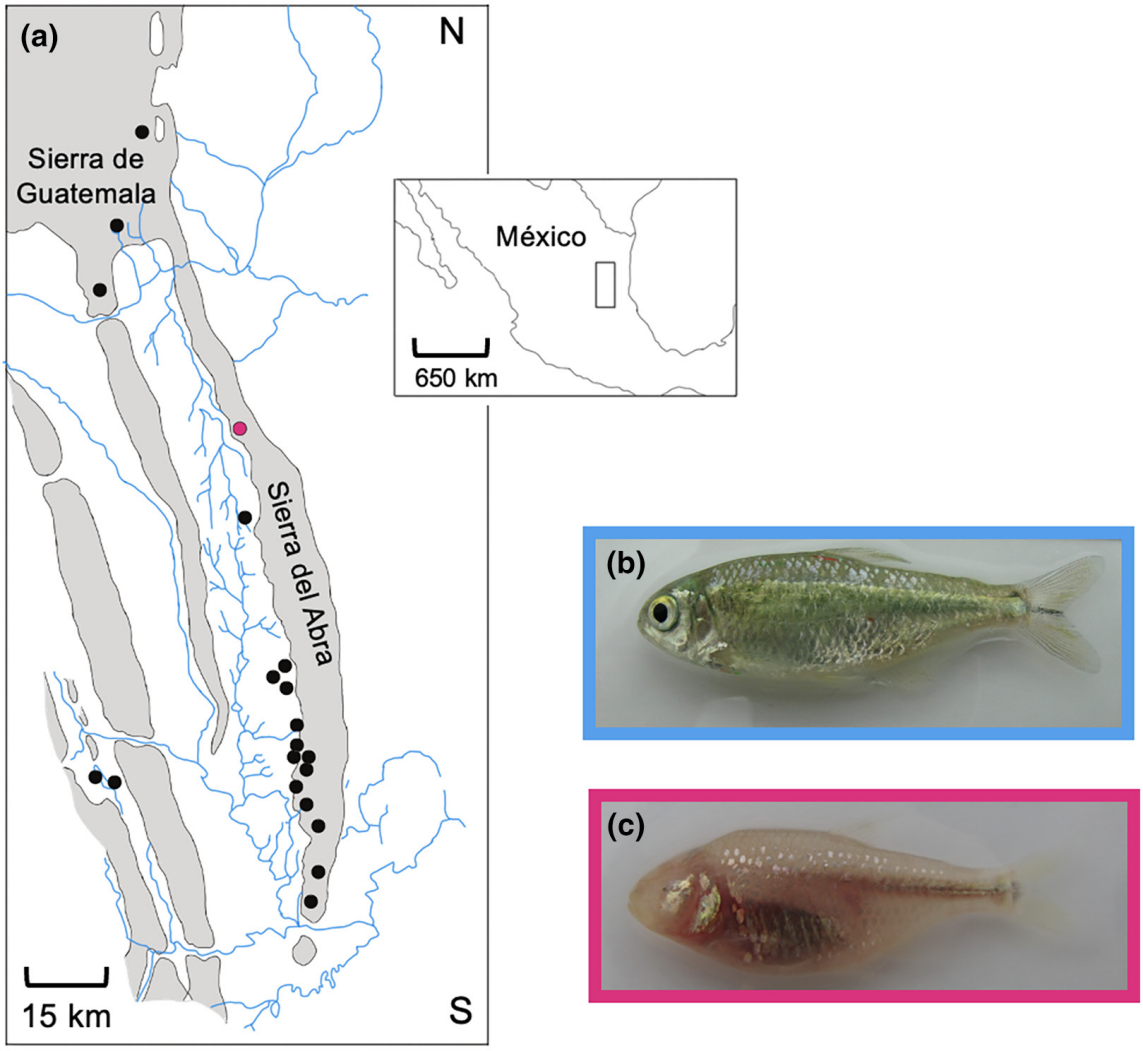
*Astyanax mexicanus* as a model for adaptive evolution. (a) Map of the karst region in México containing a subset of the caves in Sierra del Abra and Sierra de Guatemala where cavefish of this species evolved. There are at least 30 known caves housing cavefish of the Mexican tetra in this region, and not all are represented in this schematic (Mitchell et al., 1977). Blue lines represent rivers, black circles represent caves, while the magenta circle specifically represents the Pachón cave. (b) Image of an adult surface fish. (c) Image of an adult Pachón cavefish.

Vertebrate animals have also colonized caves, including salamanders and fish (Bradley, 2018; Gross, 2012). Over the past decade, one particular cavefish species, *Astyanax mexicanus*, gained a lot of traction as a novel research model to address evolutionary development of sensory systems (Figure 1) (Gross, 2012; Jeffery & Martasian, 1998; Yamamoto & Jeffery, 2000). Colloquially known as the blind Mexican cavefish, this species is particularly suitable to uncover the mechanisms underlying adaptation to the cave habitat as they have extant, river‐dwelling surface populations with intact eyes living in close proximity to the caves. During the Pleistocene, multiple independent surface fish colonizations established at least 30 geographically isolated *Astyanax* cavefish populations in Northeastern Mexico (Bradic et al., 2012; Herman et al., 2018; Gross, 2012; Mitchell et al., 1977; Ornelas‐García et al., 2008; Pérez‐Rodríguez et al., 2021; Strecker et al., 2012). After subsequent radiation underground, founder cavefish were isolated in separate caves and evolved eye regression, reduced pigmentation, and enhanced sensory systems with behavioral changes associated with survival in the caves (Gross et al., 2009; Jeffery & Martasian, 1998; Yoshizawa et al., 2014). While most studies have focused on the mechanisms underlying eye regression in this species (for a comprehensive review on cavefish eye loss, see Krishnan & Rohner, 2017), there is still a lot of opportunity to learn how other non‐visual sensory systems compensate, and whether these emerged as a direct consequence of eye loss.

Although there is constructive evolution of the olfactory and gustatory systems in *A. mexicanus* (Berning & Gross, 2023; Blin et al., 2018), this review will focus on the mechanosensory lateral line, another system that compensated for vision loss in blind cavefish (Teyke, 1990). The lateral line houses hair cell mechanoreceptors, similar to those found in the inner ear, but that are superficially distributed in round‐shaped neuro‐sensory organs called neuromasts along the body plan of the fish (Dambly‐Chaudière et al., 2003). Hair cells of the lateral line are important for detecting prey, escaping predators, and allowing fish to perform rheotaxis and other complex social interactions with conspecifics (Butler & Maruska, 2016). In zebrafish, the lateral line system has been widely studied, with numerous research groups around the world using it to understand the mechanisms underlying development and regeneration (Behra et al., 2012; Jiang et al., 2014; Lush & Piotrowski, 2014; Piotrowski & Baker, 2014; Thomas et al., 2015). Some labs have leveraged the potential of this system as a screening tool for ototoxicity (Chiu et al., 2008; Lukasz et al., 2022), while others uncovered transcriptomic signatures underlying regeneration of hair cell mechanoreceptors which are not regenerating in humans (Baek et al., 2022; Behra et al., 2012). In some populations of *A. mexicanus* cavefish, the lateral line is expanded compared to their eyed surface fish ancestor (Powers et al., 2018; Teyke, 1990). Although a number of studies addressed this expansion, mostly in the context of behavior (Jaggard et al., 2017; Lloyd et al., 2018; Yoshizawa et al., 2014), there are still a lot of unknowns regarding the emergence of supernumerary neuromasts in cavefish.

Over the last decade, a myriad of tools were developed to harness the full potential of *A. mexicanus* as a model for evolutionary biology, developmental biology, genetics, and neurobiology. A fully assembled genome for surface fish and Pachón cavefish (McGaugh et al., 2014; Warren et al., 2021), methods for transgenesis (Stahl et al., 2019), transcriptomic analyses (McGaugh et al., 2020), genetic mapping (Riddle et al., 2021), robust and reproducible behavioral assays (Chin et al., 2018; Duboué et al., 2011; Kowalko et al., 2013; Rodriguez‐Morales et al., 2022), and the application of gene editing with CRISPR/Cas9 (Klaassen et al., 2018) have positioned the field in an unprecedented scenario, with limitless possibilities. Now more than ever, we can attempt to understand the mechanisms – genetic or environmental – behind expansion of the lateral line and learn clues from this model that can ultimately inform evolutionary biology and biomedical research altogether.

In this review, I intend to discuss what is known about the morphology and developmental basis of lateral line expansions in cavefish of the Mexican tetra, commenting on their unique contributions to behavioral adaptations. I recognize, however, that this sensory system is highly variable between species, populations, and even within siblings, and that the causes of these variations are multifactorial, including additive effects of genetic and ecological contributions. Finally, I intend to highlight what I consider are a few major gaps and open avenues for future research on this system, and speculate on potential implications for evolutionary biology of sensory systems.

## 2 THE LATERAL LINE: FUNCTION AND DEVELOPMENT

The lateral line is a superficial mechanosensory system that is present in amphibian and fish species (Pichon & Ghysen, 2004). This sensory system is composed of small, mountain‐like neuro‐sensory organs called neuromasts distributed throughout the body plan of the animal, which have been extensively documented and described in zebrafish (Dambly‐Chaudière et al., 2003; Thomas et al., 2015). Neuromasts are seemingly isolated organs, each connected via inter‐neuromast cells, that have centrally located hair cell mechanoreceptors surrounded by different types of support cells. Hair cells of a neuromast are apically engulfed by a transparent cupula that surrounds the “hair bundles” of each cell (Hudspeth, 1982). These hair bundles are composed of a single non‐motile kinocilium and a stair‐step arrangement of stereocilia which are the initiators of mechanotransduction and are responsible for the establishment of hair cell polarity within the neuromast (Gillespie & Müller, 2009). Hair cells detect water currents and are thus essential for detecting moving prey, predators, and establishing social interactions between conspecifics (Klein & Bleckmann, 2015).

A hallmark of the lateral line system that has been widely studied in zebrafish is its regenerative capacity. While mammals are unable to regenerate hair cells, zebrafish, and other aquatic species have conserved their regeneration potential (Lush & Piotrowski, 2014; Thomas et al., 2015). Transcriptomic analysis of the larval zebrafish neuromast recently revealed distinct populations of support cells with unique transcriptional signatures and localizations, some of which are hair cell progenitors (Thomas & Raible, 2019).

### 2.1 Larval development of the anterior and posterior lateral line

Anatomically, in zebrafish the larval lateral line at approximately 5 days post‐fertilization houses about 50 neuromasts that are categorized as those of the anterior lateral line (head, ~30) and the posterior lateral line (trunk and tail, ~20) becomes bigger and more complex as the fish matures (Valera et al., 2021). The lateral line emerges from three placodes: (1) the posterior lateral line placode, which gives rise to the posterior lateral line primordium, (2) the anterodorsal placode, and (3) the anteroventral placode (Iwasaki et al., 2020). The latter two give rise to multiple primordia that eventually form the neuromasts of the anterior lateral line (Iwasaki et al., 2020). However, most developmental biologists using the zebrafish lateral line have focused on elucidating the mechanisms underlying development of the posterior lateral line, which emerges through sequential deposition of neuromasts from a rostro‐caudal migrating primordium (Chitnis et al., 2012; Dalle Nogare et al., 2020; Head et al., 2013; Sarrazin et al., 2010). By contrast, development of the anterior lateral line remains mostly understudied.

### 2.2 Canal and superficial neuromast classification in juvenile and adult fish

In juvenile and adult bony fishes, neuromasts are morphologically and functionally classified as canal neuromasts or superficial neuromasts (Coombs et al., 2014). Canal neuromasts are typically larger but smaller in number compared to superficial neuromasts (Blaxter, 1987; Song & Northcutt, 1991; Webb, 1989; Webb & Shirey, 2003) and are found in an epithelium at the bottom of the lateral line canals, localized in the head and trunk (Coombs et al., 2014). Because of their deeper positioning compared to superficial neuromasts, these respond to pressure differences depending on their canal position (Coombs et al., 2014). Meanwhile, superficial neuromasts have a wider distribution along the head, trunk, and tail with large variation among species, including goldfish and gobioid fishes (Schmitz et al., 2008; Asaoka et al., 2014; Puzdrowski, 1989). They can be organized as single neuromasts, grouped in lines, or clustered together on top of the skin (Coombs et al., 2014). In some species, including amphibians and non‐teleost fish, like esocoids, these are often referred to as “pit organs” or “pit lines,” which are relatively exposed compared to canal neuromasts (Lekander, 1949; Nelson, 1972).

### 2.3 Superficial neuromast expansions in cave adapted fish

Neuromast expansions in cave‐adapted fish mostly correspond to superficial, and not canal neuromasts (Soares & Niemiller, 2020). One of the most extreme lateral line hypertrophy phenotypes is found in the Northern cavefish, *Amblyopsis spelaea*, a member of the *Amblyopsidae* family of fish species that is comprised of obligate surface fish species, facultative cavefish species, and obligate cavefish species (Soares & Niemiller, 2020). *Amblyopsis spelaea* have no canal neuromasts, and their superficial neuromasts are protruded within papillae, by comparison to an obligate surface fish species of the same family, *Chologaster cornuta*, also known as the swampfish, whose superficial neuromasts are receded within the skin. This cave‐associated adaptation in the positioning of superficial neuromasts might be associated with their cave micro‐habitats (Soares & Niemiller, 2020). In rivers with intense currents or heavily vegetated habitats, maintaining neuromasts receded within the skin or within canals seemingly confers protection to these organs in surface‐dwelling fish. However, in cavefish, neuromast exposure may be an advantageous trait for surveying their surroundings, while not being as prone to damage or threats within the stillness of the cave environment.

Superficial neuromasts are expanded in cavefish species but also in highly diverse deep sea fishes and aquatic species from low‐flow habitats (Coombs et al., 1988; Denton & Gray, 1989; Marshall, 1996; Poulson, 2001; Soares & Niemiller, 2020). Although deep sea fishes are not entirely blind, and have the ability to respond to bioluminescent sources at depth, they strongly rely on their lateral line for prey detection (Johnson et al., 2009; Marshall, 1996), and do not display schooling behaviors (Cahn et al., 1968; Greenwood et al., 2013; Kowalko et al., 2013), similar to blind cavefish, and discussed here below (see Section 4).

## 3 ANATOMY OF THE CAVEFISH LATERAL LINE

**FIGURE 2:**
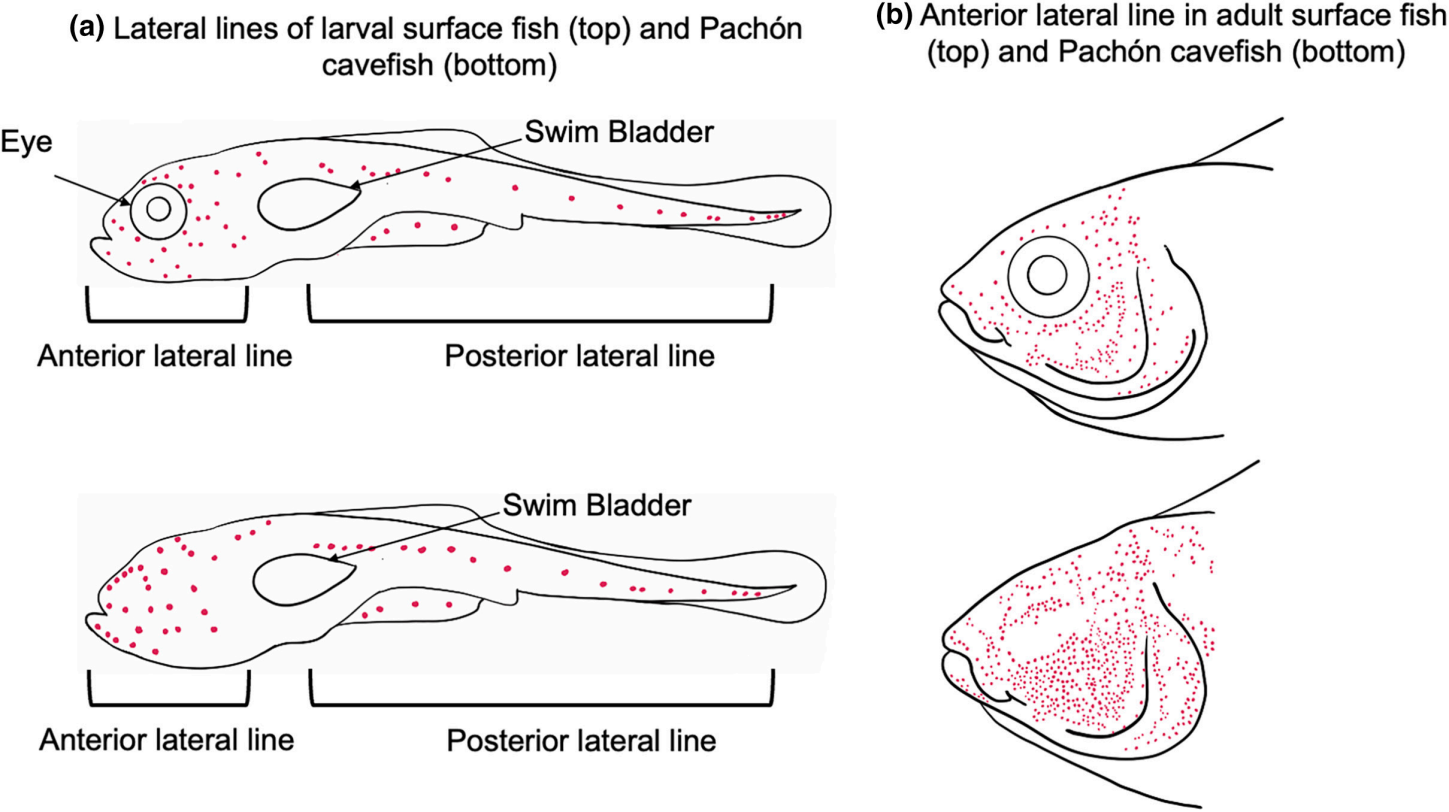
Schematic representation of the mechanosensory lateral line in *Astyanax mexicanus*. (a) Whole‐body lateral view representation of a 6‐day post‐fertilization surface fish (top) and Pachón cavefish (bottom) larva with neuromasts (red) of the anterior and posterior lateral lines (adapted from Lunsford et al., 2022). (b) Lateral view representation of the heads of adult surface fish (top) and Pachón cavefish (bottom) with neuromasts of the anterior lateral line (red) (adapted from Yoshizawa et al., 2010). These are only representations, and they do not necessarily reflect all lateral line phenotypes found across cavefish populations, which can be highly variable.

The lateral line of some, but not all, *A. mexicanus* cavefish has a major peculiarity: it is anatomically expanded when compared to its eyed, river‐dwelling surface fish ancestor (Teyke, 1990; Yoshizawa et al., 2014). These expansions are also found in other cavefish species (Soares & Niemiller, 2020). In *A. mexicanus* cavefish, lateral line expansions mostly correspond to the anterior lateral line and are apparent as early as 6 days post‐fertilization (dpf) (Figure 2) (Lunsford et al., 2022). Expansion of the lateral line means that there are more neuromasts and, in turn, more hair cells. Hair cell numbers within neuromasts are variable between cavefish and surface fish, but also between blind cavefish populations. For instance, Los Sabinos cavefish display more numerous hair cells than Pachón cavefish, which in turn have more hair cells than sighted surface fish (Teyke, 1990). Taken together, hair cell and neuromast number are highly variable traits between and within morphotypes.

Neuromast expansions in the anterior lateral line are documented within and around the eye region and have been referred to as “eye orbit” neuromasts. Eye orbit neuromasts are positioned dorsal to the infraorbital canal, where the eye would be located in sighted surface fish, making these neuromasts anatomically distinct from infraorbital neuromasts (Figure 2) (Yoshizawa et al., 2012). Because of their anatomical uniqueness, eye orbit neuromasts are associated with a cave‐evolved behavior (vibration attraction behavior, VAB), which is discussed in more detail below. These specific neuromasts are potentially a major source of lateral line information in cavefish because (1) they are expanded earlier (Lunsford et al., 2022), (2) are innervated from elsewhere and (3) have a distinct embryonic origin (Iwasaki et al., 2020).

**FIGURE 3:**
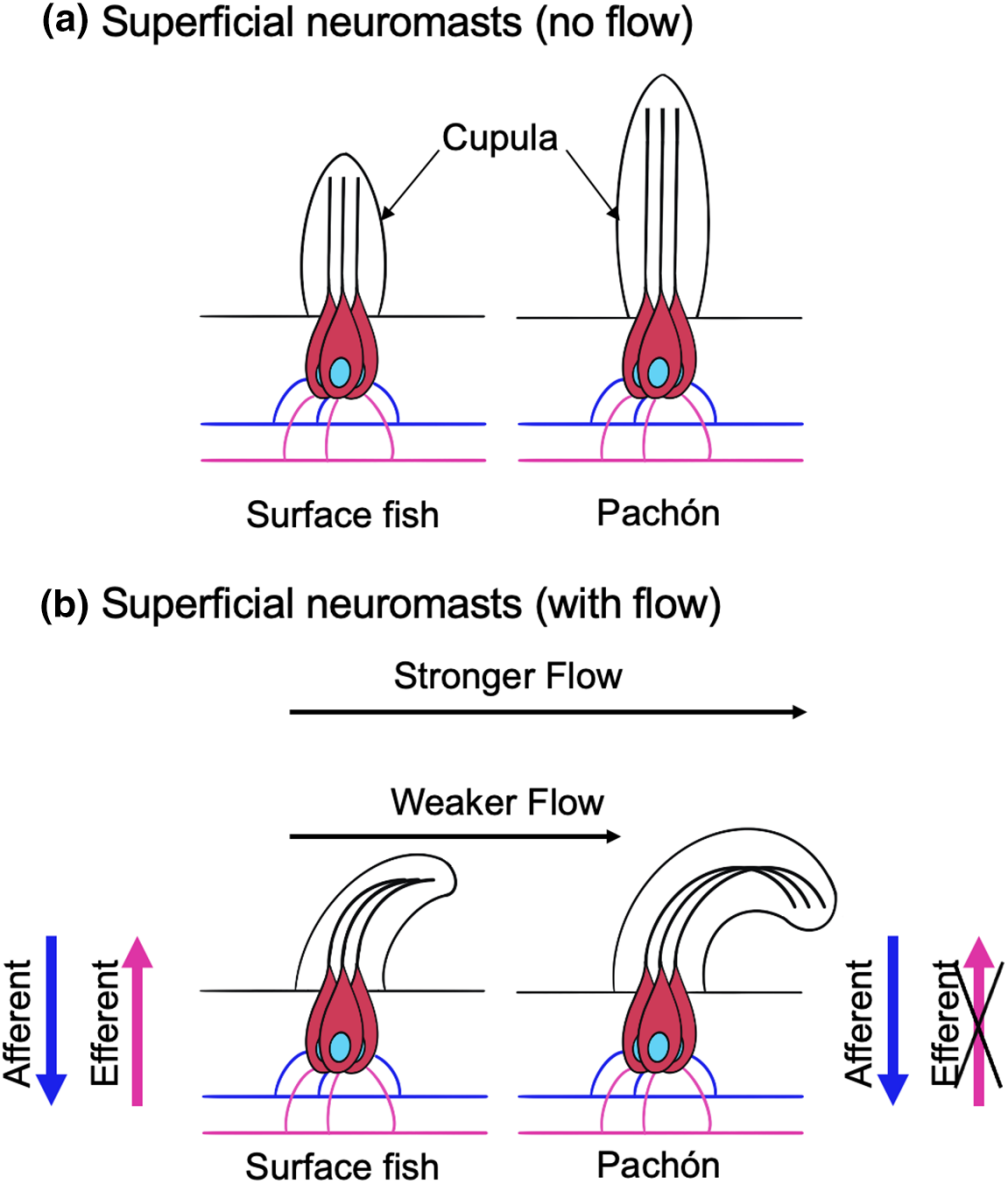
Model of the proposed contributors increasing lateral line sensitivity in blind cavefish. Schematic representation of a superficial neuromast in surface fish (left) and Pachón cavefish (right) at baseline (a) or under stimulation with water flow (b). Hair cells are represented in red. Kinocilia are represented for each hair cell, engulfed by the gelatinous cupula. Stereocilia and support cells are not represented for clarity. Each hair cell is innervated by an afferent neuron (dark blue) and an efferent neuron (magenta).

A structural paper from 1990 provided a thorough morphological examination and comparison of neuromasts in surface fish and Los Sabinos cavefish using electron microscopy (Teyke, 1990). In blind Los Sabinos cavefish (Mitchell et al., 1977; Yoshizawa et al., 2014), neuromasts were 80 × 50 μm in size, with oval basal‐shaped cupulae (Teyke, 1990). Anterior lateral line neuromasts were described as the ones with the longest cupulae (approximately 150 μm, with a maximal length of 300 μm), while posterior lateral line neuromasts had smaller cupulae (100 μm). The authors also reported a relationship between fish size and length of the cupula, with smaller fish having longer cupula compared to larger fish. In contrast, eyed surface fish were reported to have smaller neuromasts with especially smaller cupulae (approximately 40 μm, with a maximal length of 50 μm) compared to Los Sabinos cavefish. On average, Pachón and Los Sabinos cavefish had larger cupulae than surface fish (300 μM vs. 40 μm), independent of the size of the individual, helping them detect lower frequency water fluctuations (Teyke, 1990). Longer cupulae, because they extend farther out from the surface of the fish and towards stronger water currents, receive greater hydrodynamic inputs than smaller ones (see model in Figure 3b), which helps explain increased sensitivity. Utilizing these measurements, the authors propose that neuromast adaptations of the cupula may improve the fish's lateral line perception, compensating for vision loss.

## 4 LATERAL LINE AND BEHAVIOR

Expansions of the lateral line, similar to eye degeneration, have been associated with the emergence of behavioral adaptations in cavefish, including wall following, foraging, mating, sleep, schooling, and aggression. In addition to these, a seemingly “novel” behavior called “vibration attraction behavior” (VAB) emerged in cavefish and has been functionally linked to the lateral line.

### 4.1 Rheotaxis and wall following

Rheotaxis is a natural orienting behavior found in most aquatic organisms, from freshwater to benthic and pelagic fishes, where fish “orient” themselves onto an upstream or incoming water current (Arnold, 1974; Coombs et al., 2020). This behavioral response aids fish species in resisting being swept or carried away with the water flow and is accomplished by employing a variety of sensory cues, including vision (Trump & McHenry, 2013). Fish devoid of visual cues, including blind cavefish of the Mexican tetra, still exhibit innate rheotaxis behaviors (Trump & McHenry, 2013), raising the possibility that this is mediated through the mechanosensory lateral line. Although there has been a long‐standing debate over the contribution of the lateral line to rheotaxis in fish species, including cavefish, the current state of knowledge suggests that the lateral line is indispensable for the display of this innate behavior in fish (Baker & Montgomery, 1999; Janssen, 2000; Montgomery et al., 1997; Trump & McHenry, 2013; Van Trump et al., 2010). This suggests that other sensors must be at play for cavefish to efficiently perform rheotaxis in the absence of vision, including the sense of touch (Arnold, 1974; Trump & McHenry, 2013).

Although rheotaxis does not seem to be affected by the lateral line, another behavior that is associated with navigation in blind cavefish and is associated with the lateral line is wall following (Sharma et al., 2009). As in other non‐fish species that are visually deprived or restricted, *A. mexicanus* cavefish exhibit wall‐following behaviors after being introduced to a novel environment (Patton et al., 2010). While sighted surface fish do not perform wall following under lighted conditions, they display wall‐following behavior when introduced to a novel dark environment (Sharma et al., 2009). Remarkably, blocking the lateral line with a cobalt chloride treatment reduced the ability of cavefish to follow a convex wall, but not a concave or straight wall (Patton et al., 2010). An explanation for this could be a complementary mechanism between the lateral line and the sense of touch (Patton et al., 2010). While tactical sensation is the dominant sensory modality underlying wall following when the fish is very close to the wall surface, as in a concave or straight wall, the lateral line is employed when the fish is farther away from the wall, as in the case of a convex wall (Patton et al., 2010). The idea is that both senses play a role in informing the fish when it is “too close” (touch sense) or “too far” (lateral line) from the wall, allowing for an elegant cross‐talk of sensory information and higher brain processing.

### 4.2 Vibration attraction behavior (VAB)

VAB, the attraction of cavefish to a source of water vibration in a cave pool, is a constructive behavioral trait that is intimately related to the lateral line (Abdel‐Latif et al., 1990; Parzefall, 1983; Yoshizawa et al., 2010). Superficial neuromasts are associated with VAB, but not the more internal, canal neuromasts, or the hair cell containing inner ear sensory organs, as evidenced by experiments showing differences in maximal vibration frequencies that are recognized by each structure and are associated or not with VAB (Popper, 1970). This behavioral trait seems to confer an advantage only to blind cavefish, as it increases their ability to find prey in an environment with no light and low access to nutrients with a lack of macroscopic predators (Yoshizawa et al., 2010; Yoshizawa & Jeffery, 2011). In wild surface fish populations, however, VAB is suggested to have a deleterious effect, rendering them more detectable by predators (Yoshizawa & Jeffery, 2011), an important reason why surface fish would not evolve VAB.

Cavefish show the strongest VAB at 35 Hz and intermediate levels at 10 and 50 Hz, while some lab‐raised surface fish show an intermediate level VAB at a broader range (5–35 Hz) (Yoshizawa et al., 2010; Yoshizawa & Jeffery, 2011). Maximal vibration frequency is relevant for detecting animate objects in the cave pools, such as in the case of small invertebrates, including copepods, that can produce 30–40 Hz vibrations (Montgomery & Macdonald, 1987). Previous studies on the sound and vibrations emitted by water droplets showed that water droplets falling from 1 to 10 m heights can produce 40–60 Hz frequencies, which are close to the peak of detection in cavefish associated with VAB (Pumphrey & Walton, 1988). With these numbers, we can interpret that surface fish would not be great at detecting such food sources solely via the lateral line.

Interestingly, genetic mapping of VAB detected two significant quantitative trait loci (QTLs) only with the 35 Hz stimulus, suggesting that cavefish VAB at 35 Hz has a genetic basis (Yoshizawa et al., 2012). The weak VAB observed in some surface fish populations in response to a broader range is unlikely to have a genetic basis, probably resulting from developmental or environmental plasticity. In finding signaling cascades involved in the expansion of eye orbit neuromasts and VAB in cavefish, sonic hedgehog (shh) emerged as a promising candidate (Menuet et al., 2007; Yamamoto et al., 2009, 2004). Overexpression of *shh* along the anterior midline in the developing cavefish embryo underlies lens apoptosis and eye degeneration, hypothetically opening up free space for eye orbit neuromast deposition (Yamamoto et al., 2004). Because this overexpression was recently linked to jaw and taste bud expansions in cavefish, it would be reasonable to think that it also underlies expansions of the anterior lateral line (Yamamoto et al., 2009). Elegant experiments overexpressing *shh* in surface fish embryos resulted in variable eye diameters, but did not result in enhancement of the anterior lateral line at the eye orbit, or increase VAB (Yoshizawa et al., 2012). This suggested that the extra space opened up by eye regression as a consequence of *shh* signaling is not enough to promote neuromast expansions (Yoshizawa et al., 2012). The authors behind this work proposed that adaptive evolution of the lateral line contributed to eye degeneration, but it could also be that eye regression and lateral line expansions evolved independently and are controlled by different sets of genes.

### 4.3 Foraging

Food is critical for survival of a species, but finding it can be particularly challenging in the perpetual darkness of caves, where most species rely on insects, small organic compounds, or bat guano (Espinasa et al., 2017). In addition to the scarcity of food, a number of species rely heavily on visual cues for finding it, which is not an ideal mechanism in the caves. To circumvent these problems, *A. mexicanus* cavefish evolved adaptations in food‐seeking strategies, where they switched to using the lateral line for detection of moving prey (Lloyd et al., 2018). An important finding in prey‐capture behaviors in *A. mexicanus* was the differences in strike dynamics between surface fish and Pachón cavefish when exposed to live *Artemia* or brine shrimp. Surface fish performed strikes towards moving *Artemia* by bending the caudal‐most portion of their tail, in a “J” turn, while blind Pachón cavefish performed a “C” turn, bending at the level of their heads, where eye orbit neuromast expansions are present (Lloyd et al., 2018). This represented preliminary evidence that foraging evolved an anterior lateral line dependency in blind cavefish. Lateral line ablation experiments in juvenile surface fish and Pachón cavefish performed by the same group demonstrated the importance of this system for prey‐capture (Lloyd et al., 2018). Inhibiting the lateral line with the antibiotic gentamicin reduced both strike angle and strike distance towards prey in Pachón cavefish. While surface fish displayed no change in either strike angle or strike distance post‐lateral line ablation under lighted conditions, both prey‐capture measurements were zero when fish were assayed in the dark (Lloyd et al., 2018). This suggested that while surface fish do not have an expanded lateral line, they rely on it exclusively to find their prey when deprived of vision and do not fair well without it (Lloyd et al., 2018; Yoshizawa et al., 2010). While some cavefish, like Pachón cavefish, depend more on the lateral line than surface fish, they seem to be more resilient and efficient in recruiting alternative strategies to find food in comparison to surface fish. These alternative strategies might involve the recruitment of enhanced olfactory and gustatory modalities.

Further, finding food can be easier in groups, particularly when deprived of one or more sensory capabilities. Although Pachón cavefish have been described as “asocial” fish (Iwashita & Yoshizawa, 2021), with reductions in schooling and shoaling (Kowalko et al., 2013), and an absence of hierarchical social structures compared to surface fish (Elipot et al., 2013), other evidence suggests they might work in teams for efficiently finding food (Bleckmann et al., 1991). The lateral line of Pachón cavefish can be used as an alert mechanism of food availability between individuals in a shoal (Bleckmann et al., 1991). Hydrodynamics studies in *A. mexicanus* showed that Pachón cavefish in motion produce 30–90 Hz frequencies in turbulence, a signal that other cavefish can detect hypothetically for social interactions, including alerting other cavefish to the presence of food (Bleckmann et al., 1991). More recently, in a study focused on auditory perception, artificially exposing Pachón cavefish to a sound emitted by other Pachón cavefish triggered the display of a feeding posture even in the absence of olfactory cues (Hyacinthe et al., 2019). This suggested that hair cell mechanoreceptors were enough to alert the Pachón cavefish on the possibility of finding food nearby, an alert artificially triggered by Pachón cavefish sounds. Although unrelated to the lateral line, these communication mechanisms are happening through similar hair cell mechanoreceptors than those found in the lateral line, providing strong evidence in support of the importance of mechanosensory systems for the survival of blind cavefish.

### 4.4 Mating

Mating preferences are a type of social behavior that is critical for survival and adaptation of a species. In aquatic species, mating preferences are largely dependent on visual cues, including body size (Andersson & Iwasa, 1996; Ryan & Keddy‐Hector, 1992). This has been remarkably documented in Poeciliids, where female fish have a stark preference for larger males (Aspbury & Basolo, 2002; Ptacek & Travis, 1997; Rosenthal & Evans, 1998; Ryan & Wagner, 1987; Schlupp et al., 1994; Witte & Ryan, 1998). However, in subterranean environments with complete visual deprivation, mating preference cannot depend on visual assessments. Surprisingly, in *Poecilia mexicana*, also known as the Atlantic molly, cave‐adapted females display a preference for larger males in darkness, while ancestral surface female mollies do not display a preference when tested in the dark (Plath et al., 2004). This suggests that cave mollies evolved a non‐visual sensory mechanism to discriminate male size, which has been suggested to be via the lateral line (Plath et al., 2004). Whether these sex‐discrimination mechanisms are occurring in *A. mexicanus* remains an open question.

### 4.5 Sleep

Over the last decade, one of the most studied behavioral adaptations in *A. mexicanus* cavefish was sleep loss (Duboué et al., 2011, 2012; Jaggard et al., 2018, 2019; McGaugh et al., 2020). Sleep is reduced in virtually all cavefish populations of the Mexican tetra examined to date, with promising gene candidates and neural circuits already identified that might be involved in sleep adaptation (Jaggard et al., 2018; McGaugh et al., 2020). More recently, sleep loss was associated with lateral line enhancements in Pachón cavefish (Jaggard et al., 2017). Ablation of the lateral line with the antibiotic gentamicin restored sleep in adult Pachón cavefish while exerting no significant effect in adult surface fish populations that underwent the same treatment (Jaggard et al., 2017). However, although other cavefish populations have reduced sleep, like Tinaja, Los Sabinos, and Molino cavefish, only Pachón cavefish exhibited enhanced sleep when treated with gentamicin (Jaggard et al., 2017). This was puzzling, as neuromasts with enhanced mechano‐sensation, as evidenced through morphological analysis or quantification of VAB, have also been documented in Los Sabinos, Piedras, and Tinaja cavefish (Teyke, 1990; Yoshizawa et al., 2010). These findings imply that evolution of sleep loss is heterogeneous across populations while controlled by different genetic and neural mechanisms, some of which might converge with the lateral line, only in Pachón cavefish. It is also a prime example of how these troglobitic phenotypes can be highly variable between cave populations from different caves, suggesting cave‐specific ecological contributions at play.

### 4.6 Social behaviors: schooling and aggression

Adaptation of social behaviors is also present in blind cavefish populations of the Mexican tetra, including reductions in schooling and shoaling (Kowalko et al., 2013; Patch et al., 2022). One would hypothesize that expansions of the lateral line underlie reductions in schooling, potentially as a mechanism of repulsion between highly sensitive cavefish. However, previous work using surface fish‐Tinaja cavefish F2 hybrids showed no correlation between neuromast expansions and increased time spent schooling (Kowalko et al., 2013). This implies that cavefish reductions in schooling might have evolved independently from expansions of the lateral line (Kowalko et al., 2013), in line with findings in other non‐cavefish species, like *Devario aequipinnatus* (giant danios) (Mekdara et al., 2018; Tidswell et al., 2023) *Hemigrammus bleheri* (rummy‐nosed tetras) (Faucher et al., 2010; McKee et al., 2020) and *Aldrichetta forsteri* (yellow‐eyed mullets) (Middlemiss et al., 2017). It does not rule out, however, the possibility that schooling and lateral line expansions could be related to other cavefish populations.

Another behavior that evolved reductions in blind cavefish populations of the Mexican tetra is aggression (Parzefall & Hausberg, 2001). Previous work from others and I showed that multiple populations of *A. mexicanus* cavefish exhibit reductions in aggressive behaviors (Elipot et al., 2013; Espinasa et al., 2022; Rodriguez‐Morales et al., 2022). Although one might think that not seeing another individual would eliminate social interactions, in *A. mexicanus*, surface fish remain aggressive when visually deprived, while blind Molino cavefish conserved aggressive behaviors (Rodriguez‐Morales et al., 2022). Previous work in the juvenile Atlantic salmon, *Salmo salar*, showed an increase in aggression in the dark, providing evidence that light and visual cues are not necessary for the display of aggressive behavior (Valdimarsson & Metcalfe, 2001). This is in stark contrast with schooling, where studies across species show that while the lateral line is not needed for schooling, vision remains indispensable, with fish unable to school efficiently in the dark (Kowalko et al., 2013; Tidswell et al., 2023). The opposite is happening in aggression, where vision seems to be entirely dispensable for aggressive interactions to ensue among aggressive surface fish or semi‐aggressive Molino cavefish (Rodriguez‐Morales et al., 2022). This raises the very likely possibility that other, non‐visual sensory systems are recruited for conspecific detection and reaction during aggression. A strong candidate system is the mechanosensory lateral line, which has been proposed by others in the past as a contributor to the evolution of aggression (Elipot et al., 2013). To the best of my knowledge, this hypothesis has not been tested in *A. mexicanus*. However, in the African cichlid fish, *Astatotilapia burtoni*, the lateral line is an important factor in promoting motivation towards aggressive interactions (Butler & Maruska, 2015). When highly aggressive *A. burtoni* males were paired against each other either with intact lateral lines or with chemically ablated lateral lines following a cobalt chloride treatment, reductions in initial “non‐contact behaviors” that precede attacks were observed in lateral line‐ablated fish (Butler & Maruska, 2015). These reductions resulted in decreased motivation to engage in aggressive interactions, suggesting that the lateral line is used as a tool to receive and interpret positional information regarding the presence of another fish during aggressive encounters in cichlids. Interestingly, cavefish with reductions in aggression, and schooling, have more sensitive lateral lines, a seemingly opposite effect of what is described in *A. burtoni* males. One possibility is that whether fish will exhibit schooling‐like social dynamics, aggressive‐like social dynamics, or no social dynamics, like in the case of blind cavefish, will likely depend on integrative functions at higher brain regions.

## 5 SENSITIVITY OF THE CAVEFISH LATERAL LINE

The idea that the lateral line of some *A. mexicanus* cavefish populations is more sensitive than that of surface fish was mostly supported by morphological and behavioral data for many years, as described here (Teyke, 1990; Yoshizawa et al., 2014; Yoshizawa & Jeffery, 2011). Only recently empirical evidence showed that morphological expansions alone are not the sole cause of increased mechano‐sensation, but that some individual neuromast organs are more sensitive than others in the same fish, and between populations (Lunsford et al., 2022; Yoshizawa et al., 2014).

Mathematical modeling and hydrodynamics studies showed that neuromasts of the eye orbit and infraorbital neuromasts in Pachón and Los Sabinos cavefish were twice as sensitive as those of surface fish (Yoshizawa et al., 2014). The authors proposed that increased sensitivity of the cavefish neuromasts was a result of morphological enlargement of the cupula compared to surface fish (Figure 3). In a carefully designed experiment, Pachón and Los Sabinos cavefish were exposed to a vibrating rod provoking a hydrodynamic stimulus, and both populations approached it at frequencies of 35 Hz but ceased to come close at higher frequencies, while surface fish never approached it (Yoshizawa et al., 2014). These results supported the notion that neuromasts of blind cavefish are more sensitive than those of surface fish. Interestingly, the authors noted that VAB decreased in older cavefish, particularly in Los Sabinos. However, VAB extinction was not coupled to a decrease in sensitivity, as neuromasts from small and large Los Sabinos cavefish remained equally sensitive (Yoshizawa et al., 2014). Larger Pachón cavefish, however, lost VAB and decreased neuromast sensitivity when compared to smaller Pachón. The loss of VAB in spite of maintenance in neuromast sensitivity in Los Sabinos cavefish could be associated with changes in the nervous system or cavefish having learned better mechanisms for foraging through the use of olfactory or auditory cues (Menuet et al., 2007; Yoshizawa et al., 2014).

When comparing neuromast sensitivity between regions in cavefish, that is, those of the eye orbit versus those of the infraorbital region, Yoshizawa et al. (2014) also noted that only the ones from the eye orbit were associated with VAB. They proposed a heightened influence of eye orbit neuromasts on VAB could be caused by additional factors other than increased neuromast sensitivity (Yoshizawa et al., 2014). They added that the central nervous system may be more responsive to stimuli detected by eye orbit neuromasts, meaning only these are involved in the production of VAB, or foraging (Yoshizawa et al., 2014). Selective ablation of individual neuromasts would clarify or provide evidence in support of this hypothesis. With the publication of the more recent larval and adult cavefish brain atlases, performing studies on surface‐cave F2 hybrids where brain activation, neuromast number/size, and display of VAB can be quantified would shed light on this question (Jaggard et al., 2020; Kozol et al., 2023; Loomis et al., 2019).

A more recent study showed evidence that even individual hair cells in cavefish are more sensitive compared to those of surface fish by examining afferent and efferent innervation (Lunsford et al., 2022). Extracellular live recordings of afferents innervating posterior lateral line hair cells in larval surface fish and Pachón cavefish during active swimming in their basal state or in response to vibration provided evidence in support of enhanced neurotransmission in Pachón cavefish (Lunsford et al., 2022). By quantifying afferent activity before a swim bout, during a swim bout, and after a swim bout, they noticed that surface fish hair cell afferents decrease neurotransmission almost to quiescence, while Pachón cavefish only partially reduced it (Lunsford et al., 2022). Interestingly, when quantifying hair cell afferent neurotransmission across other cavefish populations, the authors found that Molino cavefish displayed an intermediate phenotype (Lunsford et al., 2022). This fits well with previous findings addressing other questions in *A. mexicanus*, where Molino cavefish have displayed intermediate phenotypes within physiological and behavioral contexts (Espinasa et al., 2022; Riddle et al., 2018; Rodriguez‐Morales et al., 2022; Xiong et al., 2018), and is another instance where ecological, cave‐specific contributions might be underlying cave‐trait variation.

Because efferent innervation at the hair cell synapse is known to have an inhibitory effect, the authors thought there was a reduction in efferent neurons of the lateral line in Pachón cavefish (Lunsford et al., 2022). By performing backfilling of cholinergic neurons in the hindbrain that innervate posterior lateral line hair cells, they showed that these were not reduced in Pachón cavefish compared to surface fish (Lunsford et al., 2022). However, when performing laser ablations of the same cholinergic neurons in both Pachón cavefish and surface fish, they found that only surface fish displayed an increase in neurotransmission, similar to the phenotype observed in Pachón cavefish (ablated or non‐ablated) (Lunsford et al., 2022). Pachón cavefish remained unaffected, with sustained neurotransmission. Inhibition of the hair cell synapse in surface fish during swimming is similar to other fish species with intact visual systems, which suggests a unique mechanism evolved in blind cavefish for sustained neurotransmission of the lateral line hair cell afferents (Flock & Russell, 1973; Lunsford et al., 2022, 2019; Pichler & Lagnado, 2020). However, because efferent neurons are still present and innervating posterior lateral line hair cells in Pachón cavefish, it is likely that other mechanisms are underlying enhanced neurotransmission, like higher sensitivity of the ribbon synapse (discussed below).

## 6 GAPS IN KNOWLEDGE

While expansions of the lateral line in dark‐adapted cavefish have been known for several years, many questions regarding its development, function, and evolutionary contribution remain unaddressed. For example, whether larger lateral lines in some species and populations of blind cavefish emerge as adaptations to the cave condition, or merely as a by‐product of the evolution of other cave‐associated traits through pleiotropy, remains unknown. Future studies looking at the contribution of eye degeneration to the lateral line phenotype in *A. mexicanus* could shed light on this question. In the past, lens ablation experiments performed in surface fish embryos were used to determine the effects of eye degeneration on cranio‐facial morphology (Dufton et al., 2012). Using a similar approach, future work could address if supernumerary neuromasts arise in surface fish lacking functional eyes following surgical lens removal during embryonic development. Similarly, surface fish mutants lacking functional eyes, like the *retinal homeobox gene 3* (*rx3*) mutant, would be an invaluable tool to determine if eye degeneration precedes lateral line expansions (O'Quin et al., 2013; Warren et al., 2021). While the most obvious hypothesis is that the lateral line expands as a consequence of eye degeneration, it could be that the visual system is impacted by signals from the early lateral line primordium. In zebrafish, the posterior lateral line primordium appears in the embryo as early as 18 h post‐fertilization (hpf), similar to the emergence of the lens placode in *A. mexicanus* (Kimmel et al., 1995; Sarrazin et al., 2010). However, eye degeneration starts in cavefish at about 40–42 hpf, a timepoint where most neuromasts of the larval fish are already deposited, both in the head and trunk (Alunni et al., 2007; Thomas et al., 2015). Thus, it is possible that molecular signals from the developing and expanding lateral line in the head trigger degeneration of the cavefish eye. Performing neuromast ablations in surface fish while the lateral line is in its early embryonic development might uncover these answers.

To answer these questions, we would need to have a better understanding of lateral line development in *A. mexicanus*. We are largely assuming similarities with zebrafish, but a lot of unknowns are currently clouding what we know about the function and contribution of the lateral line to the cave condition. A huge gap is the developmental origin of supernumerary neuromasts in cavefish with expanded lateral lines. These neuromasts may come from pre‐existing neuromasts through stitching events, as what happens in the more mature lateral line of juvenile zebrafish (Ghysen & Dambly‐Chaudière, 2007). Alternatively, and perhaps more interestingly, supernumerary neuromasts may arise from a yet unidentified and cavefish‐specific lateral line placode. This can be addressed by generating *A. mexicanus* surface fish and cavefish transgenic lines that can be used to monitor lateral line development in vivo, allowing for lineage tracing experiments. Specifically, an *A. mexicanus* equivalent to the zebrafish *Tg(cldnb:EGFP)* line, which expresses membrane‐bound GFP under the control of *claudin b* expression in all membranes of the lateral line, including the migrating posterior lateral line primordium, would be an ideal tool to uncover the developmental origin of supernumerary neuromasts in cavefish (Haas & Gilmour, 2006). The biggest piece of the puzzle, however, will be more difficult to uncover and that is the developmental and cellular origin of lateral line expansions in the head, the part that is mostly associated with cave‐adapted behavioral phenotypes (e.g. VAB and foraging). What complicates the picture is that, even in zebrafish, little is known about development of the anterior lateral line. To the best of our knowledge, only recently two anterior lateral line placodes were described in zebrafish using transgenic lines, which were referred to as the anterodorsal and anteroventral placodes (Iwasaki et al., 2020). This work also showed that not all neuromasts in the head form from migratory primordia but that some neuromasts emerge from nonmigratory, budding primordia and that emergence of some of these depends on gene expression from the hyoid region (Iwasaki et al., 2020). That is, anterior lateral line development, and potential emergence of cavefish supernumerary neuromasts, might be dependent on development of the skull. Interestingly, sensory‐skeletal integration is a phenomenon that has been studied previously in *A. mexicanus*, where changes in bone development are associated with variation in neuromast numbers (Gross et al., 2016; Powers et al., 2020). For instance, cavefish populations exhibit asymmetry between the left and right sides of their heads in fragmentation patterns of the infraorbital bone, which are positively correlated with neuromast asymmetry (Gross et al., 2016). To the best of our knowledge, no studies have looked at the contribution of candidate genes to the ontogeny of both skull and anterior lateral line in cavefish, offering a promising avenue for future research.

Another major unknown is the underlying cause behind increased neurotransmission in the cavefish lateral line. A recent study discussed in this review showed that increased neurotransmission in the cavefish lateral line is associated with a partial loss of function of efferent innervation to posterior lateral line hair cells (Lunsford et al., 2022). Efferent innervation provides a protective mechanism for the hair cell and helps regulate neurotransmission at the hair cell synapse. Neurotransmission in hair cells is tightly regulated by specialized structures called synaptic ribbons that ensure sustained and uniform delivery of glutamate‐filled synaptic vesicles at the post‐synaptic density (Lenzi & Von Gersdorff, 2001; Matthews & Fuchs, 2010; Moser et al., 2006). This regulation requires a delicate balance and coordination between pre‐synaptic and post‐synaptic components, like ribeye proteins and glutamate receptors (Lenzi & Von Gersdorff, 2001; Matthews & Fuchs, 2010; Moser et al., 2006). It is plausible that loss of function of efferent innervation in blind cavefish affects the arrangement of these pre‐ and post‐synaptic elements. Perhaps ribeye aggregates are larger in blind cavefish, recruiting larger quantities of glutamate‐filled vesicles to be released at the post‐synaptic density, ultimately contributing to increased neurotransmission. If this were the case, higher sensitivity in the cavefish lateral line could be due to cumulative effects of loss of efferent innervation, gain of function at the level of the afferent, and increased mechanotransduction caused by morphological variations of the cupula, as discussed here (See model in Figure 3b). Future work labeling synaptic ribbons in *A. mexicanus* may shed light on these questions.

Because efferent innervation at the hair cell is described as a protective mechanism against strong mechanical stimulation, one might argue that cavefish hair cells are left relatively “unprotected” from such damage. This opens up another area for future studies in cavefish, by looking at the effects of ototoxic compounds on these mechanoreceptors. Extensive studies in zebrafish have used the lateral line to screen for compounds that are damaging hair cells, including heavy metals like copper sulfate or cadmium, and antibiotics like neomycin, gentamicin, and kanamycin (Hernández et al., 2006; Owens et al., 2009; Schmid et al., 2020). Interestingly, reductions in hair cell mechanotransduction seemingly confer protection from cadmium‐induced toxicity (Schmid et al., 2020). Given increased rates of mechanotransduction and neurotransmission in cavefish hair cells, it is likely that these hair cells might also be more sensitive than those of surface fish or zebrafish to cadmium exposure. It would be interesting to see if cell death pathways, like the p53 and Bcl2 pathways, are differentially activated between hair cells of surface fish and cavefish with expanded lateral lines (Coffin et al., 2013). Taken together, *A. mexicanus* could serve as a new model for screening ototoxic compounds with increased resolution in the context of sensory divergence.

## 7 CONCLUSION

Although most evolutionary and developmental studies have focused on eye regression in cavefish, the mechanosensory lateral line stands as one of the major examples of constructive evolution in *A. mexicanus*. While several lines of work have shed light on the contribution of lateral line expansions on behavior, multiple areas of opportunity remain open to better understand this system in the context of development and synaptic plasticity. Further, the lateral line of *A. mexicanus* holds as a promising environmental and biomedical tool for ototoxic compound screening. Future work on this system will improve our understanding of constructive trait evolution, while building a bridge between ecology and evolutionary medicine.

## AUTHOR CONTRIBUTIONS

**Roberto Rodríguez‐Morales:** Conceptualization (lead); funding acquisition (lead); investigation (lead); writing – original draft (lead); writing – review and editing (lead).

## CONFLICT OF INTEREST STATEMENT

The authors declare no conflicts of interest.

## Data Availability

No original data were generated. All the supporting data are included in the references provided.
